# Effect of developmental stress on the *in vivo* neuronal circuits related to excitation–inhibition balance and mood in adulthood

**DOI:** 10.3389/fpsyt.2023.1086370

**Published:** 2023-02-10

**Authors:** Se Jong Oh, Namhun Lee, Kyung Rok Nam, Kyung Jun Kang, Kyo Chul Lee, Yong Jin Lee, Jeong-Ho Seok, Jae Yong Choi

**Affiliations:** ^1^Division of Applied RI, Korea Institute of Radiological and Medical Sciences, Seoul, Republic of Korea; ^2^Department of Psychiatry, Yonsei University College of Medicine, Seoul, Republic of Korea; ^3^Department of Radiological and Medico-Oncological Sciences, University of Science and Technology (UST), Daejeon, Republic of Korea

**Keywords:** trauma, early life stress, neurotransmission, positron emission tomography, sex

## Abstract

**Introduction:**

Traumatic events in early life have a deleterious effect on the development of normal brain developments, which may be a cause of various psychiatric disorders in adulthood. Most prior studies focused on molecular biological aspects, and research on functional changes in neural circuits is still limited. We aimed to elucidate the effect of early life stress on *in vivo* excitation–inhibition and serotonergic neurotransmission in the adulthood using non-invasive functional molecular imaging (positron emission tomography, PET).

**Methods:**

To compare the effect of stress intensity, early life stress animal models were divided into single trauma (MS) and double trauma groups (MRS). MS was derived from maternal separation, whereas MRS was derived from maternal separation and restraint stress after birth. And to evaluate the stress vulnerability on the sex, we used male and female rats.

**Results:**

The MRS group showed greater weight loss and more severe depressive/anxiety-like behaviors than the MS and control groups. Corticosterone levels in MRS showed a greater extent of decline than in the MS group; however, there was no significant difference in the change of T3 and T4 between MS and MRS. In the PET, the stress exposure groups showed lower brain uptake for GABAergic, glutamatergic, and serotonergic systems compared with the control group. The excitatory/inhibitory balance, which was derived by dividing glutamate brain uptake into GABAergic uptake, increased as stress intensity increased. Neuronal degeneration in the stress exposure groups was confirmed by immunohistochemistry. In the sex comparison, female showed the greater changes of body weight, corticosterone level, depressive/anxiety-like behavior, and neurotransmission systems than those in male.

**Conclusion:**

Taken together, we demonstrated that developmental stress induces dysfunction of neurotransmission *in vivo*, and that females are more vulnerable to stress than males.

## Introduction

Early life stress (ELS) refers to chronic physical or psychological stimuli that exceed the limitations of coping ability in childhood (1). Such stimuli include neglect of the caregiver, violence, bullying, or sexual abuse (2). Since childhood is a crucial developmental period for the maturation of neuronal circuits, the brain is sensitive to harmful stressors (3). Exposure to ELS induces dendritic spine loss, neuronal atrophy, and impaired synaptic plasticity and is associated with psychiatric disorders such as depression, anxiety, and post-traumatic stress disorder in adulthood (4, 5). From these perspectives, evaluating the effect of ELS on brain health is considered an important topic in neuroscience. To date, studies on the ELS have mainly focused on molecular biology; relatively few studies have focused on functional changes in neuronal circuits. Maternal separation (MS) causes a reduction in the number of neurons and synaptic plasticity proteins, such as postsynaptic density 95, synaptophysin, and growth-associated binding protein 43 (6). Rat dams exposed to nesting stress have hypercoticosteronemia owing to activation of the hypothalamic-pituitary-adrenal (HPA) axis, which regulates neuroendocrine adaptation for the stress response (7).

Neuronal circuits mainly comprise both excitatory and inhibitory neurons. These neurotransmissions are regulated by glutamate and gamma-butyric acid (GABA) (8). In the physiological state, the central nervous system is stabilized by balancing E/I; however, under pathological conditions, an E/I imbalance interferes with normal neural coherence (9, 10). Chen et al. demonstrated that MS reduces the frequency of excitatory postsynaptic currents, while increasing inhibitory postsynaptic currents to generate a decreasing E/I balance (11). In addition, serotonin, one of neurotransmitters, regulates mood, appetite, sleep, and arousal. Abnormalities in the serotonergic system are closely associated with various mood-related psychiatric disorders (12). However, the effect of developmental trauma on E/I balance and the serotonergic system in adulthood remains unclear.

Sex may affect various aspects of mental disorders, such as the type of disorder, severity of symptoms, and reactivity to therapeutic treatment (13–15). Although men have a high incidence of schizophrenia, autism, and attention-deficit/hyperactivity disorders than women, women are more vulnerable to depression, anxiety, and post-traumatic stress disorder (PTSD) than men (16). According to the results of the study in the same PTSD group, female patients had more severe symptoms and a longer duration of symptoms than male patients. However, to date, studies on how sex shows different neurobiological characteristics depending on ELS are insufficient.

Elucidating functional changes play an important role in broadening our understanding of the psychopathological characteristics of ELS. Since functional changes in the brain occur before structural changes, preferential evaluation of brain function is important for early diagnosis and appropriate treatment intervention. To assess brain function, positron emission tomography (PET) is frequently used to evaluate biochemical changes due to its extraordinary sensitivity. Therefore, we explored (i) the functional alterations of E/I balance as well as serotonergic neurotransmission in a developmental stress model using glutamatergic, GABAergic, and serotonergic specific PET radiotracers; and (ii) which sex is more susceptible to developmental stressors.

## Materials and methods

### Animals

All animal care and experimental protocols were performed in the same way as previously described. Sprague-Dawley rats (*n* = 6) at 2 weeks of gestation were supplied by DooYeol Biotech Co. (Seoul, Republic of Korea). Females were individually housed and checked daily before 9:00 a.m. If pups were identified, they were considered to have been born the day before [postnatal day (PND) 0], and pups were left undisturbed on this day. On PND 1, pups were cross-fostered to exclude potential effects, such as estrogen and different anxiety levels in littermates. On PND 2, the pups were randomly assigned to three groups: control (*n* = 6), MS (*n* = 6), or double trauma (maternal separation and restraint stress, MRS, *n* = 6). In the process of applying stress during the developmental stage, we chose MS as a single stressor because restraint stress inhibits animal movement itself, which sometimes induced unintended sudden death.

The care, maintenance, and treatment of animals in these studies followed protocols approved by the Institutional Animal Care and Use Committee of the Korea Institute of Radiological and Medical Sciences (IACUC permit no. KIRAMS2020-0026). Experiments involving animals were performed in accordance with the Guide for the Care and Use of Laboratory Animals published by the US National Institutes of Health. The animal housing chambers were automatically controlled at a temperature of 22 ±3°C and 55 ±20% humidity under a 12-h light/dark cycle. Sterilized rodent diet and purified tap water were provided *ad libitum*. All animal experimentations have been conducted in accordance with SfN’s Policies on the Use of Animals and Humans in Neuroscience Research.

### Stress exposure

MS stress is induced during the neonatal period. At this stage, the pups were removed from the home cage and individually placed in paper cups for 4 h (10:00–14:00) daily from PND 2 to PND 13. After separation, the pups were returned to their home cages.

**FIGURE 1:**
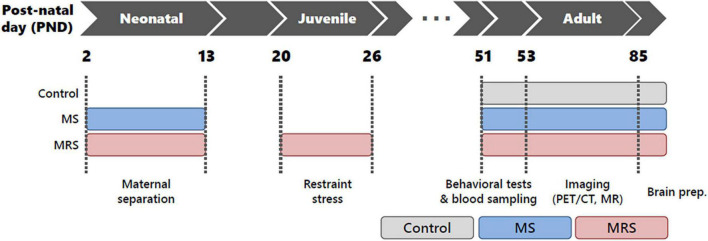
Schematic of the study protocol.

After a 1-week rest period (PND 13–19), the rats were randomly re-housed under conventional housing conditions, with two rats per cage. Restraint stress for MRS groups: the rats were restricted using an acrylic cylinder (Φ40 × 13.5 cm) for 4 h (10:00–14:00) daily from PND 20 to PND 26. After restraint, the pups were returned to their cages. The control group was not subjected to stress during the experiment. All rats were housed under standard conditions on PND 51. The study protocol is illustrated in Figure 1. Body weight was measured once a week from birth to PND 77 (1–11 weeks).

### Behavioral tests

#### Elevated plus-maze

Rats were tested in an elevated plus-maze according to previously described procedures (17). The apparatus was made of acrylic and consisted of two closed arms with walls (40 cm × 10 cm × 30 cm) and two open arms (40 cm × 10 cm) connected by an open central platform (10 cm × 10 cm). The arms were arranged such that those of the same type were opposite to one another. The apparatus was placed at the center of the room under dim illumination (30 lx) and was elevated 50 cm above the floor. The animals were individually placed on the central platform facing an open arm and allowed to freely explore the maze for 5 min. After each session, the apparatus was cleaned using 70% ethanol. Behaviors were recorded with a video camera (Hubble 300, Screen For You Co., Kyoto, Republic of Korea) mounted vertically above the apparatus, and the time spent in the open and closed arms was analyzed using video tracking software (SMART, Panlab Harvard Apparatus, Barcelona, Spain).

#### Forced swim test

This test was performed as previously described (17). Rats were placed in acrylic cylinders (20 cm diameter × 45 cm height) filled with fresh water (35 ±1 cm), and water temperature was maintained at 25 ±1°C. Rats were forced to swim for 15 min on the day before the test. After 24 h, the duration of immobility was recorded for 5 min on the test day. After each swim, the rats were carefully dried, and the water was changed between the experiments to ensure that the rats were clearly visible. The immobility time was recorded using a commercial software (SMART, Panlab Harvard Apparatus, Spain).

### Thyroid hormones and corticosterone

To measure thyroid hormones (T3 and T4) and corticosterone, blood samples were collected on the day of the forced swim test. Blood samples were directly collected into serum separator tubes (BD Vacutainer^®^ SST™, Becton-Dickson, ON, Canada) from the tail vein of the awake rats and left for 30 min at room temperature. After centrifugation at 1,500 *g* for 20 min at 4°C, serum was separated from whole blood and stored frozen at −70°C before the ELISA experiments. To examine hormone levels, serum was diluted according to each reaction condition (T3, T4 = 1:4; CORT = 1:3) and reacted with the rat thyroid hormone magnetic bead panel for T3 and T4 (no. RTHYMAG-30K-03) and rat stress hormone magnetic bead panel for corticosterone (no. RSHYMAG-69K-02) overnight at 4°C (Milliplex MAP kit, EMD Millipore, MA, USA). After the reaction, the samples were analyzed using a Luminex (Luminex, TX, USA) and repeated in triplicate.

### Molecular imaging

#### Preparation of radiotracers

To evaluate alterations in brain circuits, including the dopaminergic, glutamatergic, and serotonergic systems, three different types of radiotracers were used. [^18^F]flumazenil, [^18^F]FPEB, and [^18^F]Mefway are specific radiotracers for GABAergic (i.e., GABA_A_ receptor), glutamatergic (i.e., mGluR5), and serotonergic systems (i.e., 5-HT_1A_ receptor), respectively. The radiotracers were prepared according to a previously described procedure (17, 18). The radiochemical purity of all radiotracers at the end of the synthesis was over 95%.

#### PET/CT scan

Brain PET images were acquired using a small-animal PET scanner (nanoScan^®^, Mediso, Budapest, Hungary). The scanner has a peak absolute system sensitivity greater than 9% in the 250–750 keV energy window, an axial field of view of 28 cm, a transaxial field of view of 35–120 mm and a transaxial resolution of 0.7 mm at 1 cm from the center. Each animal was anesthetized with 2.5% isoflurane in oxygen, and [^18^F]flumazenil, [^18^F]FPEB, and [^18^F]Mefway PET imaging was performed at intervals of 2 days (15.52 ±1.3*MBqofradioactivitiesintoteilvein*). As all PET scans were performed on the same animal at 2 days intervals, the possibility of mutual interference between radiotracers was eliminated. To avoid the possible effect of differences in molar activity, all PET scans were performed alternately between the experimental and control groups. In the case of [^18^F]Mefway, aqueous fluconazole (60 mg/kg, OneFlu injection, JW Pharmaceutical, Republic of Korea) was pre-administered for 1 h to overcome the spill-over from skull radioactivity. A 40–60 min static acquisition was performed because all radiotracers showed that specific-to-nonspecific binding values reached a plateau after 40 min. The image scans were acquired in an energy window of 400–600 keV. Images were reconstructed using a 3D ordered subset expectation maximization (3D-OSEM) algorithm with four iterations. For attenuation correction and anatomical reference, micro-CT imaging was conducted immediately after PET using 50 kVp of X-ray voltage with 0.16 mAs.

#### MR scan

To define the anatomical volume of interests (VOIs), MR scans were obtained on a 31-cm horizontal-bore Agilent 9.4 T scanner (Agilent Technologies, Santa Clara, CA, USA) using a four-channel array rat head surface coil (Rapid Biomedical GmbH, Rimpar, Germany). The image parameters for the 3D turbo spin-echo (TSE) T2-weighted images were as follows: repetition time (TR) = 2,500 ms; echo time (TE) = 7.45 ms; field of view = 20 mm × 20 mm × 10 mm; matrix size = 128 × 128 × 64; voxel size = 156 μm × 156 μm × 156 μm; echo train length = 43; and scan time = 1 h 54 min 50 s. During imaging, the respiratory rate of the rats was monitored using an MR-compatible physiological monitoring and gating system (SA Instruments, Inc., Stony Brook, NY, USA).

#### Image analysis

The 3D VOI was determined to compare regional PET uptake in all groups. A study-specific brain template was used to analyze the rat brain data. Each MR image was spatially normalized to the W. Schiffer T2-weighted rat brain MRI template using PMOD software (version 3.8, PMOD Technologies Ltd., Switzerland). Normalized brain MR images were summed, and then a Gaussian filter (full width at half maximum = 1.0 mm) was applied to minimize the bias induced by noise. Depending on the characteristics of each PET tracer, the VOIs were defined on the MRI template (Supplementary Figure 1). The decay-corrected regional radioactivities were acquired from VOIs. For comparing radioactivity between inter-subject, standardized uptake value (SUV) was used to normalize for differences in the injected dose and body weight. The SUV values obtained for each region of activity were multiplied by the weight of the rat divided by the injected dose.

### Immunohistochemistry

After imaging, the rats were anesthetized with 3% isoflurane and intracardially perfused with 0.1% heparin normal saline (JW Pharm., Republic of Korea) and 4% paraformaldehyde buffer (Biosesang, Republic of Korea) to wash out the blood from the whole brain and pre-fixation. Subsequently, the brains were extracted and post-fixed with natural buffered formalin (10%), and staining was performed at the Korean Pathology Technical Center (Cheongju, Republic of Korea). Briefly, the brain samples were processed using an automatic tissue processor for 14 h (TP1020; Leica Microsystems, Wetzlar, Germany). Brain tissues were then embedded in paraffin blocks and cut into 4-μm sections using a microtome (Shandon Finesse ME Microtome, Thermo Fisher Scientific, USA). After dewaxing and rehydration, endogenous peroxidase activity was blocked with a peroxidase-blocking solution for 10 min (S2023, DAKO, Denmark).

To examine stress-related changes in NeuN immunoreactivity in the rat hippocampus, we performed immunohistochemical staining and quantitative analysis, according to a previously described method. We incubated with anti-NeuN antibody (diluted 1:100; Abcam, UK) at 4°C overnight, followed by incubation with secondary antibodies (Envision kit, DAKO, Denmark) at room temperature for 30 min; then, we washed and incubated at room temperature for 30 min with a Rabbit/Mouse Target retrieval solution (S2369, DAKO, Denmark). After that, the sample was washed, and brain tissues were stained with 3,3′-diaminobenzidine substrate for 3 min (EnVision Detection System, DAKO, Denmark) and counterstained with Mayer’s hematoxylin (Sigma-Aldrich, USA). To determine NeuN immunoreactivity in the rat hippocampus, we captured digital images using a microscope (CX31, Olympus, Japan). The image was adjusted to a magnification filed (×100), and the three areas (100 × 100 μm) were defined as the subregions of the hippocampus [CA1, CA2, CA3, and dentate gyrus (DG)]. NeuN immunoreactivity was manually counted using image-viewing software (Motic VM 3.0, Motic Microscopes, Canada).

### Statistical analysis

Quantitative results are expressed as means ± SDs. All statistical analyses were performed using the Prism software (GraphPad Software, Inc., La Jolla, CA, USA). Student’s *t*-tests were used to determine statistical signifiance at a 95% confidence level, and a value of *p* < 0.05 was considered significantly different.

## Results

### Body weight

**FIGURE 2:**
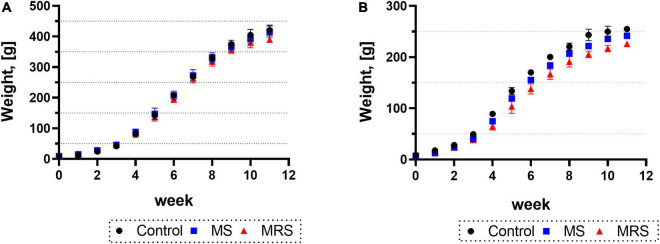
Comparison of the body weights in control, MS, and MRS group [**(A)** male and **(B)** female]. Data are presented as means ± SD (*n* = 6).

Body weights for both males and females were similar to those at 2 weeks after birth (PND 14); however, the weight difference began to occur after 3 weeks (PND 21) (Figures 2A, B). At 11 weeks (PND 77), the MS group was 1–7% declined body weight compared to the control group (*p* = 0.5417 for male and *p* = 0.0800 for female). At the same time point of PND 77, the MRS group displayed a 9–13% weight loss compared to the control group (*p* < 0.0001 for male and *p* < 0.0001 for female).

### Behavioral tests

**FIGURE 3:**
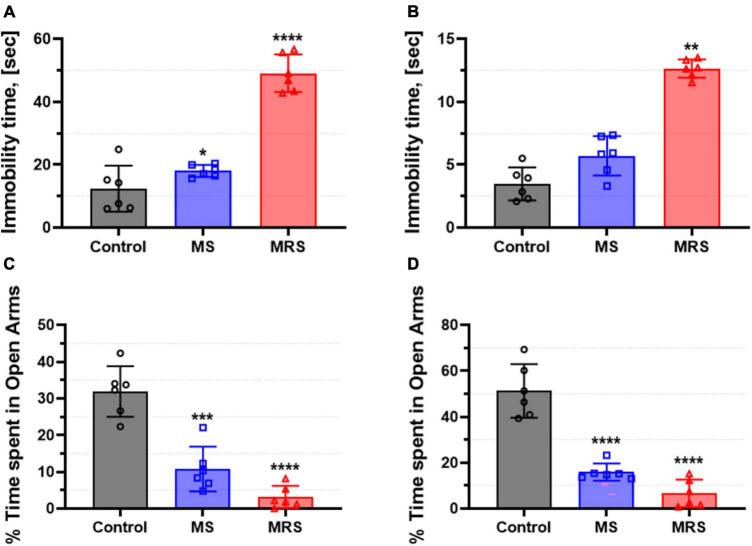
Comparison of immobility time in the forced swim test [**(A)** male and **(B)** female] and % time spent in open arms in the elevated plus-maze [**(C)** male and **(D)** female]. Data are presented as means ± SD (*n* = 6). Statistical significance was defined as a *p*-value less than 0.05 (*p < 0.05, **p < 0.01, ***p < 0.001, ****p < 0.0001).

At PND 51, forced swim tests were performed to assess depression-like behavior (Figures 3A, B). The male MS group showed 136% higher immobility time and the female MS displayed 153% increased immobility time than the control group (*p* = 0.0218 for male and *p* = 0.3353 for female). The immobility time in the MRS group increased by 229–296% compared to that in the control group (*p* < 0.0001 for male and *p* = 0.0034 for female).

Rats were tested in an elevated plus-maze to assess anxiety-like behavior (Figures 3C, D). The MS groups spent 64–66% lower in the open arms than the control groups (*p* = 0.0001 for male and *p* < 0.0001 for female). In addition, the time spent in the open arms in the MRS group was 85–86% lowered (*p* < 0.0001 for male and *p* < 0.0001 for female).

### Neuroendocrine hormones

**FIGURE 4:**
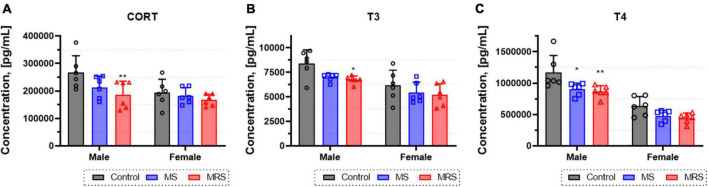
Concentration of corticosterone **(A)**, T3 **(B)**, and T4 **(C)** levels form blood serum. Data are presented as means ± SD (*n* = 6). Statistical significance was defined as a *p*-value less than 0.05 (*p < 0.05, **p < 0.01).

To assess changes in the levels of thyroid hormones and corticosteroids, the concentrations of corticosterone (CORT), triiodothyronine (T3), and thyroxine (T4) in the serum were determined (Figure 4). In the MS group, the CORT level was 5.5–20% lowered than that in the control group (*p* = 0.0619 for male and *p* = 0.6986 for female). The differences in T3 and T4 hormone levels between the MS and control group was 12–17 or 23–24%, respectively (T3: *p* = 0.0512 for male and *p* = 0.2935 for female; T4: *p* = 0.0121 for male and *p* = 0.1221 for female). The concentration of CORT was 14–30% lowered than that in the control group (*p* = 0.0082 for male and *p* = 0.3498 for female). In addition, T3 and T4 hormone levels decreased almost 15–20 or 26–30%, respectively (T3: *p* = 0.0258 for male and *p* = 0.1894 for female; T4: *p* = 0.0049 for male and *p* = 0.0590 for female).

### NeuroPET

#### Glutamate PET

**FIGURE 5:**
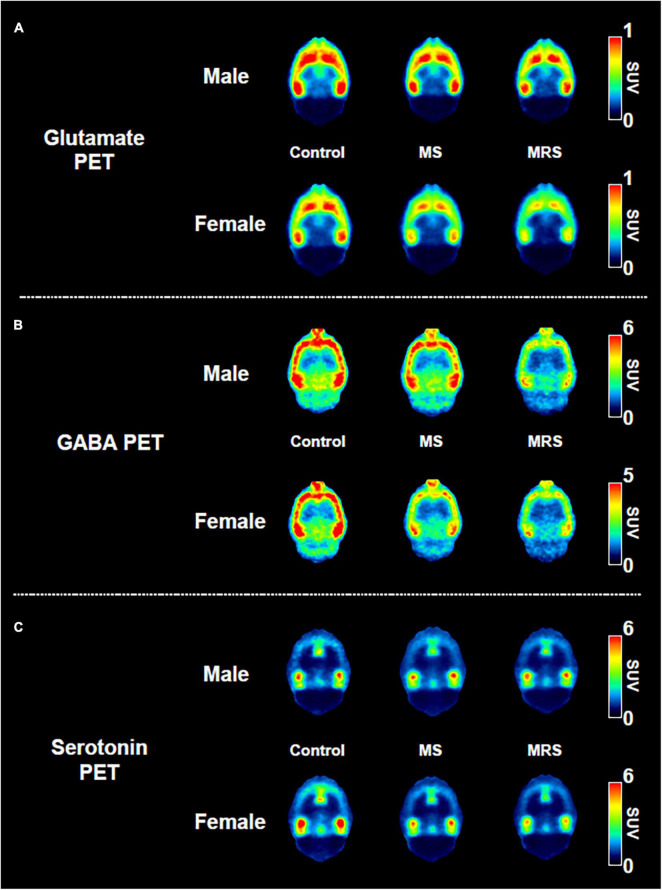
Comparison of the neuroreceptor PET images of glutamate **(A)**, GABA **(B)**, and serotonin. **(C)** The mean PET images were obtained from 40 to 60 min and categorized as male and female.

**FIGURE 6:**
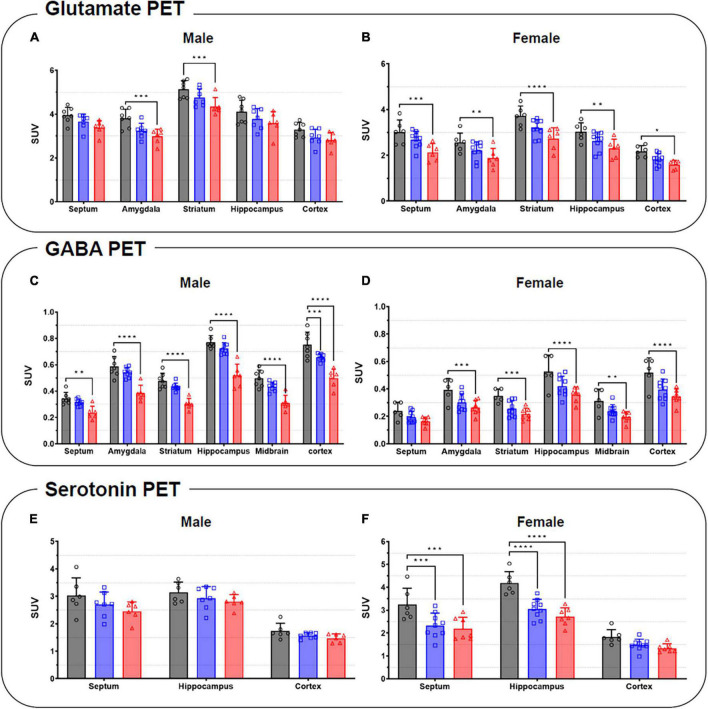
Quantification of the radioactivities for the glutamate **(A,B)**, GABA **(C,D)**, and serotonin PET **(E,F)** in the specific brain regions in male (left) and female (right). Data are presented as the mean ± the SD (*n* = 6). Statistical significance was defined as a *p*-value less than 0.05 (*p < 0.05, **p < 0.01, ***p < 0.001, ****p < 0.0001).

Summed glutamate PET images are shown in Figure 5A, which reveal that the stress exposure groups showed lower brain uptake than the control group. In the cortex, MS and MRS showed 11–28% lower radioactivity than the control group (MS: *p* = 0.0984 for male and *p* = 0.0791 for female, MRS: *p* = 0.0350 for male and *p* = 0.0151 for female; Figures 6A, B and Table 1). In the septum, the radioactivity in the MS and MRS groups was 7–32% lower than that in the control group (MS: *p* = 0.1721 for male and *p* = 0.0108 for female, MRS: *p* = 0.0137 for male and *p* = 0.0003 for female). The mean SUVs of the amygdala for the MS and MRS were 15–28% lower than those for the control group (MS: *p* = 0.0092 for male and *p* = 0.0845 for female, MRS: *p* = 0.0005 for male and *p* = 0.0045 for female). The radioactivity in the hippocampus was 8–26% lower in the MS and MRS groups than the corresponding values in the control group (MS: *p* = 0.1214 for male and *p* = 0.0511 for female, MRS: *p* = 0.0222 for male and *p* = 0.0029 for female). The striatal SUVs for the MS and MRS were 7–29% lower than those for the control group (MS: *p* = 0.0883 for male and *p* = 0.0108 for female, MRS: *p* = 0.0009 for male and *p* < 0.0001 for female).

**TABLE 1:** Comparison of the regional brain uptake values (SUV).

Neurocircuits	Gender	Group	Regional brain uptake (SUV)				
			Cortex	Septum	Amygdala	Hippocamps	Striatum
Glutamatergic system	Male	Control	3.30 ± 0.32	3.95 ± 0.36	3.81 ± 0.41	4.12 ± 0.52	5.13 ± 0.39
		MS	2.94 ± 0.38	3.66 ± 0.34	3.24 ± 0.35	3.78 ± 0.48	4.76 ± 0.40
		MRS	2.81 ± 0.35	3.39 ± 0.32	2.99 ± 0.33***	3.59 ± 0.52	4.35 ± 0.40***
	Female	Control	2.23 ± 0.23	3.14 ± 0.49	2.62 ± 0.44	3.14 ± 0.32	3.83 ± 0.38
		MS	1.82 ± 0.25	2.69 ± 0.36	2.22 ± 0.38	2.63 ± 0.39	3.19 ± 0.38
		MRS	1.60 ± 0.19*	2.14 ± 0.37***	1.88 ± 0.43**	2.31 ± 0.40**	2.73 ± 0.47****
GABAergic system	Male	Control	0.75 ± 0.10	0.34 ± 0.05	0.59 ± 0.08	0.77 ± 0.05	0.48 ± 0.06
		MS	0.66 ± 0.03	0.31 ± 0.03	0.54 ± 0.04	0.72 ± 0.05	0.43 ± 0.03
		MRS	0.50 ± 0.07****	0.24 ± 0.05**	0.39 ± 0.06****	0.52 ± 0.09****	0.30 ± 0.04****
	Female	Control	0.52 ± 0.11	0.24 ± 0.06	0.39 ± 0.08	0.53 ± 0.12	0.35 ± 0.05
		MS	0.39 ± 0.08	0.19 ± 0.04	0.30 ± 0.06	0.42 ± 0.07	0.26 ± 0.06
		MRS	0.34 ± 0.06****	0.16 ± 0.03	0.26 ± 0.05***	0.36 ± 0.05****	0.22 ± 0.04***
Serotonergic system	Male	Control	1.73 ± 0.28	3.03 ± 0.65	N.D.	3.15 ± 0.38	N.D.
		MS	1.57 ± 0.11	2.70 ± 0.46	N.D.	2.94 ± 0.42	N.D.
		MRS	1.48 ± 0.15	2.46 ± 0.34	N.D.	2.81 ± 0.25	N.D.
	Female	Control	1.86 ± 0.35	3.37 ± 0.72	N.D.	4.23 ± 0.55	N.D.
		MS	1.48 ± 0.26	2.33 ± 0.54***	N.D.	3.05 ± 0.43****	N.D.
		MRS	1.34 ± 0.19	2.19 ± 0.50***	N.D.	2.71 ± 0.39****	N.D.

Values are presented as the mean ± SD. Statistical significance was defined as a *p*-value less than 0.05 (*p < 0.05, **p < 0.01, ***p < 0.001, and ****p < 0.0001 compared to the control group, *n* = 6, N.D.: not determined). SUV, standardized uptake value.

#### GABA PET

As shown in the comparative mean PET images, brain GABA uptake in the stress exposure groups was lower than that in the control group (Figure 5B). The cortical uptakes for MS and MRS were 13–34% lowered than those of the control group (MS: *p* = 0.0007 for male and *p* = 0.0024 for female; MRS: *p* < 0.0001 for male and *p* < 0.0001 for female; Figures 6C, D and Table 1). In the limbic area, including the septum, amygdala, and hippocampus, the stress exposure groups showed 6–34% lower uptake compared to the control group, while the MS and MRS showed 8–32% lower radioactivity in the septum than the control group (MS: *p* = 0.2668 for male and *p* = 0.2589 for female, MRS: *p* = 0.0038 for male and *p* = 0.0597 for female). In the amygdala, the MS and MRS showed 7–34% lowered uptake compared with the control (MS: *p* = 0.1075 for male and *p* = 0.0306 for female, MRS: *p* < 0.0001 for male and *p* = 0.0017 for female). In the hippocampus, MS and MRS showed 6–32% lower uptake than the control group (MS: *p* = 0.0704 for male and *p* = 0.0085 for female, MRS: *p* < 0.0001 for male and *p* < 0.0001 for female). The mean SUVs of the striatum in the MS and MRS group were 10–37% lower than those in the control group (MS: *p* = 0.0863 for male and *p* = 0.0207 for female, MRS: *p* = 0.0001 for male and *p* = 0.0012 for female). In the midbrain, the uptake in the MS and MRS showed 13–38% lower radioactivity compared to the control group (MS: *p* = 0.0236 for male and *p* = 0.0664 for female, MRS: *p* = 0.0001 for male and *p* = 0.0038 for female).

#### Serotonin system

The averaged serotonergic PET images are shown in Figure 5C, indicating that the stress exposure groups showed lower brain uptake than the control group. In the cortex, the PET uptake of the MR and MRS was 9–28% lower than that of the control group (MS: *p* = 0.4689 for male and *p* = 0.1618 for female, MRS: *p* = 0.2437 for male and *p* = 0.0619 for female; Figures 6E, F and Table 1). The septal SUVs of the MS and MRS were 11–35% lowered than the corresponding values in the control group (MS: *p* = 0.1676 for male and *p* = 0.0006 for female, MRS: *p* = 0.0134 for male and *p* = 0.0002 for female). The hippocampal radioactivities of the MS and MRS were 7–36% lowered than those of the control group (MS: *p* = 0.3632 for male and *p* < 0.0001 for female, MRS: *p* = 0.1278 for male and *p* < 0.0001 for female).

### NeuN immunoreactivity

**FIGURE 7:**
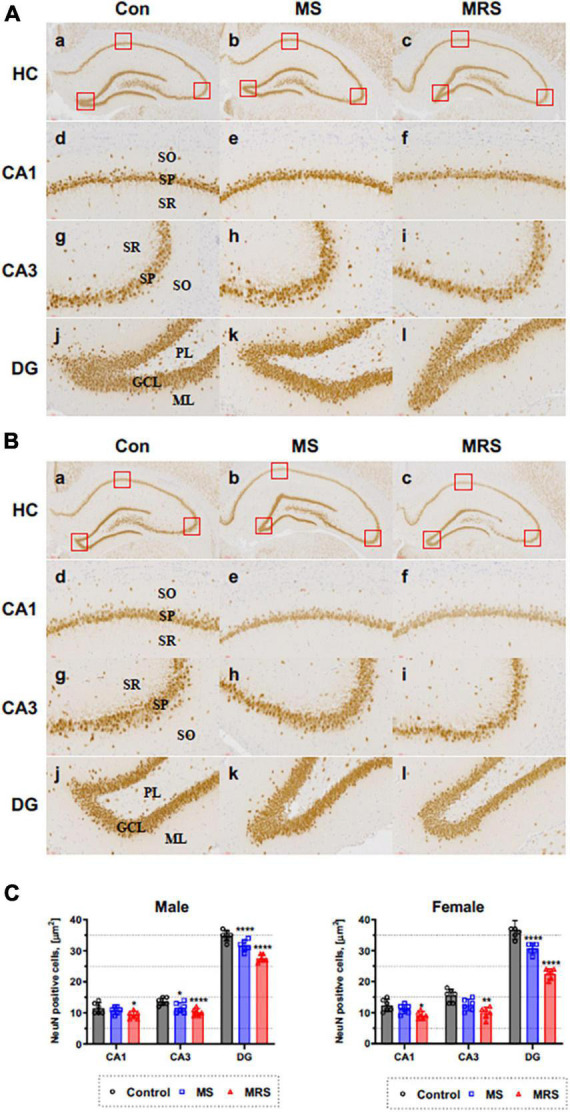
Immunohistochemical staining of NeuN in the brain of male **(A)** and female **(B)** rats in the control, MS, and MRS groups. The NeuN immunoreactive (brown color) revealed on all hippocampus region (GCL, granule cell layer; ML, molecular layer; PL, polymorphic layer; SO, stratum oriens; SP, stratum pyramidale; and SR, stratum radiatum) [a–c: hippocampus (HC), d–f: CA1, g–i: CA3, j–l: dentate gyrus (DG) (count area: 100 × 100 μm, a,b: ×100, c–h: ×400)]; **(C)** Comparison of % relative number of NeuN staining cells in the CA1, CA3, and DG region; data are presented as means ± SD (*n* = 6). Statistical significance was defined as a *p*-value less than 0.05 (*p < 0.05, **p < 0.01, ****p < 0.0001).

We measured the density of neurons to evaluate the effect of traumatic experience using the neuronal marker NeuN. Figure 7 shows that NeuN immunoreactive cells can be detected in the stratum pyramidale (SP) of CA1 and CA3 region and in the granule cell layer (GCL) of DG region.

The MS group showed 6–15% lower immunoreactivity compared with the control group (*p* = 0.3354 of CA1, *p* = 0.0174 of CA3, *p* < 0.0001 of DG for male *p* = 0.4654 of CA1, *p* = 0.0343 of CA3, *p* < 0.0001 of DG for female). For MRS group, the positive of NeuN cell 19–26% lower compare with control group (*p* = 0.0109 of CA1, *p* < 0.0001 of CA3, *p* < 0.0001 of DG for male and *p* = 0.0122 of CA1, *p* = 0.0002 of CA3, *p* < 0.0001 of DG for female).

## Discussion

We determined the effect of developmental trauma on *in vivo* neurotransmission using non-invasive functional molecular imaging. Among the stressed groups during the developmental period, the brain uptake for GABAergic, glutamatergic, and serotonergic systems was lower than that in the control group. The rate of decrease in brain uptake was proportional to stress intensity. The uptakes difference between trauma and control group in sex for GABAergic system was not evident, whereas it was confirmed that females had significantly lower binding profiles than males for the glutamatergic and serotonergic systems. The excitatory/inhibitory balance, which was derived by dividing glutamate brain uptake into GABAergic uptake, increased as stress intensity increased. The ELS experience groups showed a loss of neuronal cells in the hippocampus. Taken together, we demonstrated that developmental stress induces dysfunction of neurotransmission *in vivo* and that females are more vulnerable to stress than males. To our knowledge, this is the first study to show that developmental stress causes an E/I balance *in vivo*.

Stress in early childhood, when neural circuits develop, adversely affects normal physical and mental development compared to adulthood (19, 20). The stress exposure group showed a loss of body weight in proportion to the stress level, which was relatively greater in females than in males. These results suggest that ELS could accelerate weight loss, especially in females. In other words, compared to the control group, the weight change for single or double stressors was much greater for females than for males. This may be owing to the degree of activation of neuroendocrine systems such as the hypothalamic-pituitary-adrenal (HPA) or hypothalamic-pituitary-thyroid (HPT) axis. Chronic stress induces dysfunction in the HPA/HPT axis, which promotes eating habits (21). The corticosterone concentration associated with the activity of the HPA axis in the double stress group showed a greater decline than in the single stress group, whereas there was no significant difference in the change in thyroid hormone levels between the groups. This indicates that the HPA axis is more susceptible to developmental stressors than the HPT axis. We also observed that double stress group (MRS) showed more severe depressive/anxiety-like behaviors than the single stress group (MS). These results imply that the stronger the psychological and physical stress intensity in early life, the more severe behavioral abnormalities are induced in adulthood.

Glutamate and GABA are the most abundant neurotransmitters that control excitatory and inhibitory signals, respectively (22). These neurotransmitters play a crucial role in regulating the balance between excitation and inhibition (E/I) of neuronal activity. E/I imbalance causes psychiatric disorders; therefore, maintaining the E/I balance is crucial for mental health. We evaluated E/I balance by measuring brain uptake in glutamatergic and GABAergic PET. First, glutamate uptake was significantly lower in the MRS group than in the control group. Among the brain regions, the amygdala had the largest reduction rate, indicating that the amygdala was most vulnerable to stress regarding the excitatory system. In clinical studies, patients with depression have also shown lower glutamatergic uptake in the cortex and amygdala (23–25). Second, in the GABAergic system, the MRS group showed significantly lower radioactivity than the control group. Among the brain regions, the hippocampus showed the highest reduction rate, indicating that the hippocampus is the most sensitive tissue to stress in the inhibitory system. This PET result is in good agreement with Martisova’s work, in which maternally separated rats showed lower hippocampal GABA concentrations than the control groups (26). Third, as the stress intensity increased, the E/I ratio increased (Table 2). However, in the rate of change in E/I balance compared with the control, female was relatively lower than that for male (Table 3). This is because there was no sex difference in PET uptake in the inhibitory system, but the excitatory system showed lower PET uptake in female than in male. Collectively, it seems that major changes in E/I balance involve more excitatory systems than inhibitory systems. Serotonin systems are involved in modulating mood, cognition, reward, learning, and memory (27). Abnormalities in the serotonin system are related to stress-related disorders, such as anxiety and depression. In the present study, we evaluated ELS in the serotonergic system in adulthood. The MRS group showed a significant decrease in serotonergic uptake compared to the control group, particularly in the hippocampus. This result coincides with previous findings, in which lower serotonin expression was observed in animals exposed to stress (28–30).

**TABLE 2:** Comparison of the regional E/I ratio values.

Sex	Group	E/I value				
		Cortex	Septum	Amygdala	Hippocamps	Striatum
Male	Control	4.42 ± 0.57	11.61 ± 1.54	6.15 ± 0.63	5.34 ± 0.59	10.80 ± 1.00
	MS	4.51 ± 0.59	11.75 ± 1.68	6.30 ± 0.23	5.46 ± 0.81	11.08 ± 1.30
Female	MRS	5.77 ± 1.22	14.87 ± 3.32***	7.85 ± 1.29	7.14 ± 1.80	14.53 ± 1.62****
	Control	4.48 ± 1.19	13.54 ± 2.45	6.94 ± 1.76	5.51 ± 0.75	11.04 ± 1.27
	MS	4.51 ± 0.67	13.90 ± 1.69	6.80 ± 1.13	5.99 ± 0.62	12.05 ± 1.83
	MRS	4.85 ± 0.73	14.47 ± 1.85	7.69 ± 2.13	6.66 ± 1.19	13.23 ± 3.22

Values are presented as the mean ± SD.

Statistical significance was defined as a *p*-value less than 0.05 (***p < 0.001 and ****p < 0.0001 compared to the control group, *n* = 6).

**TABLE 3:** The rate of change in E/I balance compared to the control group.

Sex	Group	E/I value ratios compared to the control group				
		Cortex	Septum	Amygdala	Hippocamps	Striatum
Male	MS	1.03 ± 0.19	1.03 ± 0.23	1.07 ± 0.14	1.06 ± 0.17	1.04 ± 0.21
	MRS	1.30 ± 0.30	1.30 ± 0.31	1.34 ± 0.17	1.32 ± 0.37	1.37 ± 0.18
Female	MS	1.01 ± 0.30	0.62 ± 0.57**	0.96 ± 0.23	1.05 ± 0.08	1.01 ± 0.12
	MRS	1.13 ± 0.36	1.04 ± 0.23	1.12 ± 0.48	1.22 ± 0.39	1.21 ± 0.43

Values are presented as the mean ± SD.

Statistical significance was defined as a *p*-value less than 0.05 (**p < 0.01 compared to the male, *n* = 6).

Among the neurotransmission systems examined in this study, females showed lower brain uptake than males in the glutamatergic and serotonergic systems; however, there were no significant differences between females and males in the GABAergic system. The reduction rate of brain uptake was the largest in the serotonergic systems. This is probably related to the differences in sex-specific serotonin synthesis rates. Many previous studies have suggested that serotonin synthesis mechanisms are more sensitive in females than in males; therefore, major depression is higher in women than men (31–34).

A limitation of the present study is the absence of biochemical information (i.e., metabolic rate and food intake-related hormones) that supports the effect of stress on changing body weight. In addition, since only restraint stress was used for a single trauma in the present study, the effect of other traumatic events is unknown. Our previous work has demonstrated that there is no significant difference between the two groups when evaluated in adulthood, after imposing single stressors such as MS or restraint stress during the developmental periods (17). However, sexual differences in MS were not explained because the study used only males. These issues need to be addressed in a future study.

## Conclusion

Developmental stress causes weight loss, neuroendocrine system dysfunction, and depressive/anxiety-like behaviors in adulthood. This ELS leads to a reduction in neurotransmission related to the excitatory-inhibitory and serotonergic systems *in vivo*. The excitatory/inhibitory balance increased as stress intensity increased. In addition, we found that stress caused neuronal degeneration in immunoassays. In the sex comparison, females were more vulnerable to stress than males.

## Data availability statement

The original contributions presented in this study are included in this article/Supplementary material, further inquiries can be directed to the corresponding author.

## Ethics statement

The animal study was reviewed and approved by the Institutional Animal Care and Use Committee of the Korea Institute of Radiological and Medical Sciences.

## Author contributions

SO and JC contributed to conceptualization, data curation, and review and editing. JC and YL contributed to funding acquisition. NL contributed to investigation. KN, KK, KL, and J-HS contributed to methodology. SO, NL, and JC wrote the original manuscript. All authors had read and agreed to the published version of the manuscript.
