# Supplementary material for: Effect of developmental stress on the in vivo neuronal circuits related to excitation–inhibition balance and mood in adulthood
Source: Front Psychiatry. 2023 Feb 10;14:1086370. doi: 10.3389/fpsyt.2023.1086370 (PMC9950095; doi:10.3389/fpsyt.2023.1086370)
Supplement: Supplementary file 1 [file Data_Sheet_1.docx]

**Effect of developmental stress on the in vivo neuronal circuits related to excitation–inhibition and mood in adulthood**

**Se Jong Oh^1^, Namhun Lee^1^, Kyung Rok Nam^1^, Kyung Jun Kang^1^, Kyo Chul Lee^1^, Yong Jin Lee^1^, Jeong-Ho Seok^2^, Jae Yong Choi^1,3*^**

^1^Division of Applied RI, Korea Institute of Radiological and Medical Sciences, Seoul, Korea

^2^Department of Psychiatry, Yonsei University College of Medicine, Seoul, Korea

^3^Radiological and Medico-Oncological Sciences, University of science and technology (UST), Seoul, Korea

*** Correspondence:**

Jae Yong Choi

[smhany@kirams.re.kr](mailto:smhany@kirams.re.kr)

**Figure S1.**
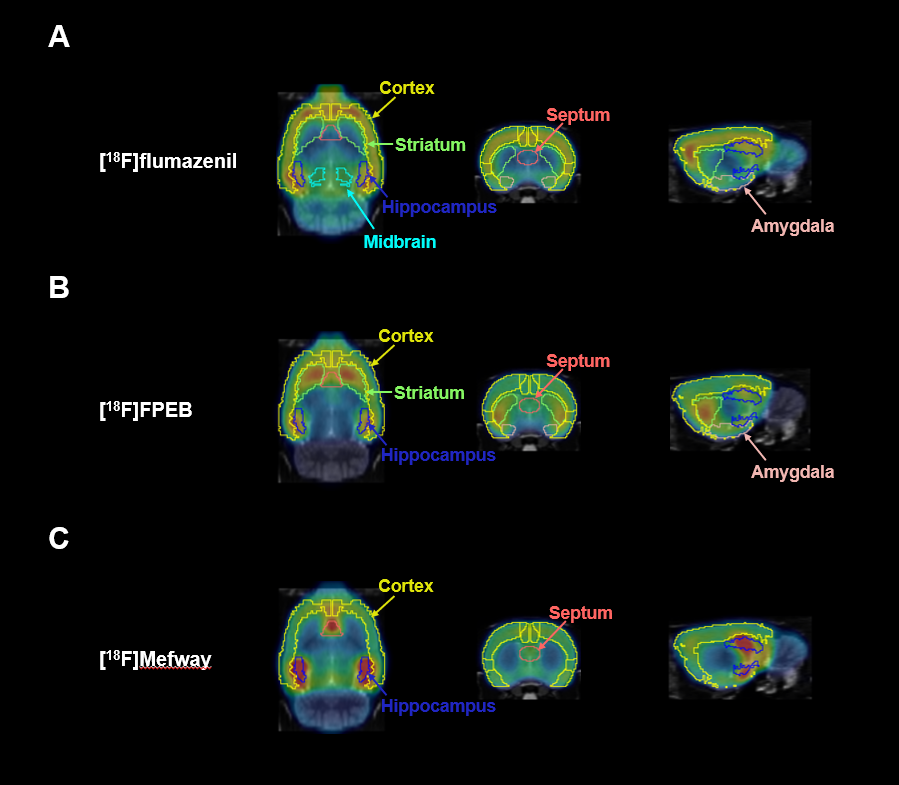


Figure S1. Definition of VOIs for [^18^F]FPEB (A), [^18^F]flumazenil (B), and [^18^F]Mefway (C). VOI applied PET images were produced using the PMOD fusion tool (version 3.8).
